# Error baseline rates of five sample preparation methods used to characterize RNA virus populations

**DOI:** 10.1371/journal.pone.0171333

**Published:** 2017-02-09

**Authors:** Jeffrey R. Kugelman, Michael R. Wiley, Elyse R. Nagle, Daniel Reyes, Brad P. Pfeffer, Jens H. Kuhn, Mariano Sanchez-Lockhart, Gustavo F. Palacios

**Affiliations:** 1 Center for Genome Sciences, United States Army Medical Research Institute of Infectious Diseases (USAMRIID), Fort Detrick, Frederick, Maryland, United States of America; 2 Integrated Research Facility at Fort Detrick (IRF-Frederick), National Institute of Allergy and Infectious Diseases, National Institutes of Health, Fort Detrick, Frederick, Maryland, United States of America; University of Malaya, MALAYSIA

## Abstract

Individual RNA viruses typically occur as populations of genomes that differ slightly from each other due to mutations introduced by the error-prone viral polymerase. Understanding the variability of RNA virus genome populations is critical for understanding virus evolution because individual mutant genomes may gain evolutionary selective advantages and give rise to dominant subpopulations, possibly even leading to the emergence of viruses resistant to medical countermeasures. Reverse transcription of virus genome populations followed by next-generation sequencing is the only available method to characterize variation for RNA viruses. However, both steps may lead to the introduction of artificial mutations, thereby skewing the data. To better understand how such errors are introduced during sample preparation, we determined and compared error baseline rates of five different sample preparation methods by analyzing *in vitro* transcribed Ebola virus RNA from an artificial plasmid-based system. These methods included: shotgun sequencing from plasmid DNA or *in vitro* transcribed RNA as a basic “no amplification” method, amplicon sequencing from the plasmid DNA or *in vitro* transcribed RNA as a “targeted” amplification method, sequence-independent single-primer amplification (SISPA) as a “random” amplification method, rolling circle reverse transcription sequencing (CirSeq) as an advanced “no amplification” method, and Illumina TruSeq RNA Access as a “targeted” enrichment method. The measured error frequencies indicate that RNA Access offers the best tradeoff between sensitivity and sample preparation error (1.4^−5^) of all compared methods.

## Introduction

Describing the genomic composition of intra-host virus populations is becoming crucial for understanding disease progression, determining the effect of immune pressure on evolution of viral genotypes and phenotypes, optimizing vaccine design, and identifying virus genome mutations that may lead to resistance against medical countermeasures [1–5]. The characterization of viral genomic populations is especially important for RNA viruses, which due to their relatively short genomes have short replication times. Due to these short replication cycles and the error-prone viral RNA-dependent RNA polymerase, RNA viruses typically are subject to extreme evolutionary dynamics with very high mutation rates, thereby leading to large population sizes [6].

Next-generation sequencing (NGS) technologies have had a dramatic impact on the experimental analysis of the genomic diversity of RNA viruses. NGS allows sampling the entire genomic sequence space of an RNA virus population in parallel during a single experiment. Nevertheless, genomic sequence characterization of viral populations *in vivo* remains challenging. For instance, separating real RNA virus variant populations from background and error noise is difficult. In addition, it is challenging to increase assay sensitivity to study samples with low viral genomic concentrations in the presence of relatively abundant host biome nucleic acids.

The need for precise variant calling algorithms for the detection of single nucleotide polymorphisms (SNPs) in mammalian genomes led to the development of correction methods for sequence errors generated during NGS [7–19]. However, identification and control of errors generated during sample preparation has received less attention. In general, RNA virus population characterization begins with viral nucleic acid extraction from samples. The isolated viral RNA population then has to reverse transcribed, followed by polymerase chain reaction (PCR) amplification of the resulting viral DNA population. Amplification (or another enrichment procedure) has to be performed because current NGS technologies require high quantities of input DNA, and because *in vivo* RNA viral genome concentrations are usually several orders of magnitude lower than the NGS detection threshold. Reverse transcriptases are error-prone RNA-dependent DNA polymerases because they lack of proof-reading functions. Therefore, reverse transcription-derived errors are unavoidable. Because reverse transcription-derived errors are introduced during the first step of sample preparation, they quickly become fixed during downstream PCR amplification and are therefore very difficult to distinguish from real nucleotide variations in RNA virus genome populations. Likewise, DNA-dependent DNA polymerases, used for PCR, may introduce additional nucleotide changes during amplification due to their intrinsic amplification error rates. Dependent on the exact amplification strategy, even more errors could be introduced. These error sources include PCR primer synthesis mistakes, biased amplification due to PCR primer mismatches, resampling errors due to very low abundance of input DNA representing particular areas of the RNA virus genome, DNA fragmentation errors depending on the length of the obtained amplicon, and generation of genomic chimeras in multiplex PCR.

Several pre-processing algorithms have been developed to remove sequence errors *in silico*. Most of these algorithms are included as standard tools in various publicly available software packages [11–13]. Some commonly used cleaning algorithms are: adaptor and primer removal, terminating base quality trimming, and removal of reads with low sequence complexity or the presence of ambiguous base calls. In addition, methods to remove reads and read pairs that do not properly describe the expected input molecules are also available. These more computationally expensive methods typically require alignment or iterative analysis to identify errors, particularly PCR duplicates and chimeric sequences.

Methods have also been developed to reduce the amount of error generated in the process of sample preparation. An elegant example of this is circular resequencing (CirSeq), which decreases error rates to only 7.6 x 10^−9^ errors/site/copy [20]. For this method, individual viral RNAs in a sample are amplified by rolling-circle reverse transcription. Consequently, each individual RNA is repeatedly reverse-transcribed and each new transcript is covalently connected to the previous one, resulting in linear tandem repeats. Alignment of the individual, redundant transcript repeats then allows the establishment of a consensus sequence, thereby correcting the majority of sample preparation and instrument error *in silico* [21]. Other sample preparation methods that can achieve lower error rates try to limit additional amplification steps and directly sequence viral RNA after reverse transcription by increasing the concentration of viral material. These enrichment strategies typically use targeted probe enrichment or host RNA depletion [22–24].

Here we determined and directly compared error rates for different NGS pre-processing methods in routine use for the characterization of intra-host RNA virus genome populations. To establish baseline error rates, we used a recombinant plasmid as a source of Ebola virus RNA and analyzed the resulting viral RNA with common methods. Since plasmid DNA propagation has a much lower error rate (≈10^−7^–10^−8^ errors/site/copy) than RNA virus genome replication [25], the plasmid-generated “viral” RNA can be considered a homogenous starting population. Consequently, genomic nucleotide variation observed after NGS are in all likelihood errors introduced by the sample preparation method, which allows a direct comparison of error rates of various methods. Using this strategy, we compared CirSeq [21], sequence independent single-primer amplification (SISPA) used in many pathogen identification and characterization applications [26, 27], targeted amplicon sequencing used in viral population genetics of rare populations [28, 29], and an in-solution capture enrichment method (TruSeq RNA Access) [3].

## Material and methods

### Sample preparation

**Fig 1:**
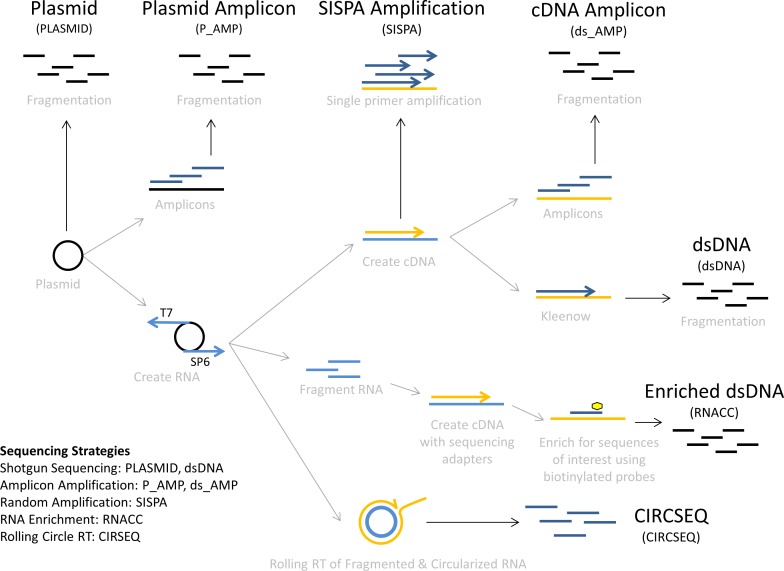
Study sample diagram.

We analyzed seven different libraries using at least two independent sample preparations (Fig 1):

1. Library “PLASMID”: a pGEM3 plasmid encoding the Ebola virus/H.sapiens-tc/COD/1976/Yambuku-Mayinga RNA-dependent RNA polymerase L, used as a surrogate system for viral nucleic acid, was transformed in MDS™42 LowMut Electrocompetent cells (Scarab genomics, Madison, WI). A single colony was picked and grown in LB medium using standard conditions. Plasmid DNA was isolated with the PowerPrep HP Maxiprep System (Origene Technologies Inc., Rockville, MD) and the resulting DNA was prepared using whole shotgun procedures;
2. Library “dsDNA”: *In vitro* transcribed RNAs were obtained from the pGEM3 plasmid above using T7 (positive stand) and SP6 (negative strand) RNA polymerases contained in the MAXIscript kit (Life Technologies, Grand Island, NY). The resulting RNAs were reverse-transcribed using the Superscript III first-strand synthesis system using the random hexamers supplied with the kit [30]. Second strand synthesis was performed using a DNA polymerase I Klenow fragment (New England Biolabs, Ipswitch, MA) and then prepared for NGS with standard whole shotgun procedures;
3. Libraries “P_AMP” and “DS_AMP”: the pGEM3 plasmid and the cDNA obtained above were used as templates for amplicon amplification using Phusion Hot Start Flex DNA polymerase (New England Biolabs, Ipswich, MA) and touchdown PCR with a panel of specific primers (sequences available upon request). The used set of primers provides double-coverage of the Ebola virus *L* (RNA-dependent RNA polymerase) gene segment to minimize primer amplification bias artifacts. The overlap length was set to ≈500 bp, and the target amplicon length was set to ≈1,200 bp. To remove fragments <500 bp, amplicons were pooled and purified using a 0.6x AMPure XP bead size (Beckman Coulter, Indianapolis, IN). The resulting pool of PCR amplicons were sequenced after DNA fragmentation;
4. Library “SISPA”: T7 and SP6 *in vitro* transcribed RNAs were reverse-transcribed using a tagged random hexamer and then amplified using a single primer as previously described [27] using MyTaq DNA Polymerase (Bioline, Tauton, MA);
5. Library “RNACC”: EBOV-specific reads were enriched from *in vitro* T7 and SP6 RNA polymerase-transcribed RNA using the Illumina TruSeq RNA Access (RNACC) kit with modifications to the manufacturers procedures described previously [3]. Fifty pg (≈4.67 x 10^6^ copies) or 1 ng were used;
6. Library “CIRSEQ”: traditional reverse transcription was replaced with CirSeq as described in [20, 21].

All library input materials, except those for RNACC, were fragmented using the Covaris S2 instrument (Covaris, Woburn, MA) and were prepared with the Illumina TruSeq DNA sample Preparation Kit (Illumina, San Diego, CA) according to the manufacturers’ protocols. All libraries were evaluated for quality using the Agilent 2100 Bioanalyzer (Agilent, Santa Clara, CA). After quantification by real-time PCR with the KAPA qPCR Kit (Kapa Biosystems, Woburn, MA), all libraries were diluted to 2 nM.

All libraries were sequenced on an Illumina MiSeq desktop sequencer using version 2 300 cycle kits (2x150). Samples were multiplexed using dual indexes (Illumina, San Diego, CA) to reduce intrarun index misassignment [31]. A coverage depth of ≈1,000x redundancy was targeted and obtained, except for CIRSEQ (≈600x).

### Data analysis

Data analysis was performed with the in-house software pipeline VSALIGN (USAMRIID, Frederick, MD). VSALIGN is a Perl wrapper for the efficient and consistent cleaning, alignment, and analysis of genetic variants for RNA virus stock characterization and sample RNA virus genome population analysis using common next-generation sequencing practices. VSALIGN combines custom code and commercially and publicly available software to automate and standardize the steps of our chosen methodology. Where possible, the software is optimized for massively parallel, resource intensive operation to increase performance.

#### Preprocessing sequencing reads

The cleaning module of VSALIGN was used to pre-process sequencing reads/read pairs to exclude sequencing artifacts and low quality data. Additionally, the module was used to segregate data that may be biologically relevant but did not match the expected Ebola virus input molecule. Many of the basic quality concerns were handled by the open-source PRINSEQ-lite software package [12]. The pre-processing steps included: adapter removal, exclusion of paired ends with index quality restriction <30 Phred; removal of PCR exact duplicates in excess of three standard deviations of the mean; identification and removal of chimeric reads or read pairs; removal of non-viral sequences; removal of read pairs with non-opposing directionality; removal of sequences of general quality (<20 Phred); masking of low complexity regions using the DUST algorithm implemented in Prinseq [12]; and trimming of primer sequences detected at the read termini. Additionally, read ends were anchored by a base with a quality score Phred >15 or they were trimmed until this condition was met.

The removal of chimeric reads listed above was more computationally expensive as alignment was required. Removal was achieved by a stand-alone parallelized implementation of the local alignment tool BLAST+ (National Center for Biotechnology Information (US); http://www.ncbi.nlm.nih.gov/). To maximize the yield and maintain cost efficiency, multiple samples were run simultaneously (“multiplexing”) by using adapter indices. Although the indices chosen are usually selected for their disparate sequences to facilitate removal of indexing errors, index misassignment between samples still occurs. Index misassignment is of great concern for large projects that must detect subclonal variants at very low frequency. To minimize this effect, we used dual indexing, a method that reduces index misassignment by incorporating an additional index sequence at the 3′ end of the read [31]. The tradeoff is the amount of useful sequence garnered. Despite this approach, we still detected cases of index misassignment. Thus, a second method was implemented by only including reads with a very high average index quality, thereby minimizing the opportunities for improper binning of reads during de-multiplexing. This methodology reduced index misassignment in both single and double index formats [31].

PCR duplicates are also a source of errors in subclonal diversity estimation. These artifacts arise from differences in amplification efficiencies or amplification bias arising from very low abundance of input material [32]. PCR single-read duplicates represented 28% of the reads on the basis of similarity or alignment to the template. However, when paired end sequencing data were used, only 8% of the read pairs were determined to be exact duplicates [33]. We therefore only eliminate PCR exact duplicate read pairs from our analysis. Since there is a significant probability that multiple viral fragments with exactly the same sequence are present in the sample, limiting the number to any particular replicate value would be deleterious to the proper estimation of minor allele abundance. As a compromise between the two approaches, we eliminated exact duplicate read pairs to more than the three standard deviations of the mean number of replicates per read across the sample.

After these cleaning steps, the majority of the reads and read pairs typically provides good quality sequence data. However, a small percentage of the reads still do not describe the intended input sequence and typically are chimeric reads (as high as 1.9% in a study of an HIV-1 population [34]). Chimeric reads and read pairs have one part of the read or pair matching the RNA virus and the second matching an unrelated sequence (e.g., adapters used in creation of the sequence libraries or even an unidentified sequence). A variety of sources are likely candidates for the origination of chimeric sequences, including, but not limited to: PCR artifacts, errors in adapter ligation, or sequence read-through. Thus, although computationally expensive, our pipeline used a similarity-based approach for the total elimination of suspicious paired end reads.

Finally, primer synthesis errors and primer bias amplification are concerns specific to targeted PCR amplification procedures. Primer sequence errors correlate with poor sequence quality. Therefore, common practice is to trim or remove primer-bearing reads. Although length/size exclusion strategies are used during primer synthesis, nucleotide misincorporations and insertions and deletions (indels) still occur at a higher rate during primer synthesis than the rate of errors observed during NGS sequencing. Identification of primer-originated sequence is straightforward due to their incorporation at the termini of the amplicons, and the sequences are typically trimmed from reads used for alignment. Primer bias on the other hand is a more insidious error. This bias occurs when a pleomorphic site in the primer-binding site results in varying amplification efficiencies, which results in misrepresentation of downstream proportions of the viral population. To overcome this type of error, we used multiple 500-bp overlapping amplicons to cover the Ebola virus sequence. The potential effect of primer selection bias was minimized by covering each base of the viral sequences with two independent amplicons (excepting at the termini).

#### Alignment and variant calling

Viral sequence assemblies were completed in DNAStar Lasergene nGen (Madison, WI). Default parameters used during the alignment matches those described previously [1, 2]. Briefly, we aligned reads that were at least 93% identical over the read (≈10-base mismatch). SNPs with fewer than 200-read depth were removed from analysis. A consensus change is defined here as a change relative to the published sequence of Ebola virus/H.sapiens-tc/COD/1976/Yambuku-Mayinga (RefSeq accession #NC_002549) present in ≥50% of the population. Below that threshold, SNPs were considered subclonal substitutions and part of a minority subpopulation of the “virus.” Alignment files for all the libraries assessed here are available at Bioproject PRJNA326412 and NCBI SRA project number SRP076923.

## Results

We compared sequencing errors due to RNA virus nucleic acid amplification and enrichment using different sample preparation methods. In particular, we analyzed the type and origin of errors after analysis of seven different libraries using two independent preparations (Fig 1).

**Fig 2:**
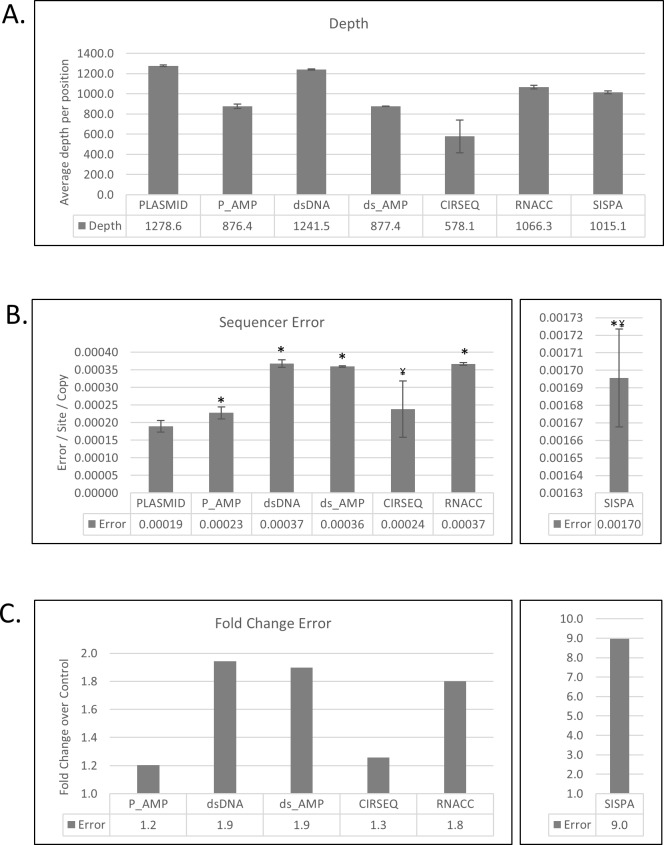
Total error by sample preparation method. (A) The mean read depth per position from two DNA controls (PLASMID and P_AMP) and five RNA sample preparation methods (n = 2 of replicate experiments). (B) Errors calculated as error/site/copy (plasmid or transcript) are presented as the average of duplicate experiments as in A. Intra-host single nucleotide variants present in the original plasmid were removed from the calculation of the error (reference positions: 4,697 and 4,725). Student t-tests were performed to demonstrate differences between the mean error per site per copy in the control plasmid (* = *p*<0.05) and each sample preparation method. (C) The error calculated in B is converted to fold change over the control (“PLASMID”). Error bars in all panels represent standard deviation of the mean.

Many post-processing algorithms rely on obtaining extreme sequencing depth to strengthen the statistical framework of the analysis. However, *in vivo*, these sequence depths are difficult to achieve due to low viral loads and abundance of host RNA. Based on empirical data, a target average depth of 600 was used for allocating the amount of sequence depth for each sample, and only positions of the genome with a minimum depth of 200 were incorporated in the analysis. In addition, the VSALIGN algorithm normalizes read depth in order to reduce the extreme peaks, based on random sampling, in overrepresented sequences due to loading or sensitivity issues. Average read depths per position are reported in Fig 2A.

The first step after cleaning and variant calling was to stratify the detected SNP positions by pattern. The initial goal was to determine which SNPs were present in the originating sample. Two sites contained two substitution intra-host single nucleotide variants (iSNVs) at percentages greater than 1% of the population in both “PLASMID” replicates (reference positions: 4,697 and 4,725). Consequently, these sites were removed from the calculation of mean error rates as the diversity was considered part of the originating sample.

The library-specific diversity was calculated as the mean error/site/copy (Fig 2B). Student t-tests were performed to demonstrate differences between the mean error per site per copy in the control plasmid (* = *p*<0.05) and each library-specific diversity, as well as between dsDNA (shotgun sequencing of the cDNA generated from the *in vitro* transcribed RNA) and the other after reverse transcription preparations (¥ = *p*<0.05). To facilitate assessment, the values were also expressed as number of fold-change between the mean error of each preparation and the plasmid control (Fig 2C). Compared to “PLASMID”, we observed that “P_AMP” resulted only in a minor mean error increase (1.2–fold), whereas “dsDNA,” “DS_AMP,” and “RNACC resulted in significantly increased acquired errors (1.8–1.9-fold), although no significant differences were observed between those libraries. As expected, “SISPA” resulted in the highest error increase (≈9.0-fold) and “CIRSEQ” the lowest error of the post reverse transcription preparations (1.3-fold). Nevertheless, whereas “CIRSEQ” error was lower in our hands to every other RNA protocol, we did not achieve the levels of fidelity measured by the inventors of CirSeq [20], probably due to our lack of expertise in the method. The “CIRSEQ” outcome was not statistically different from the least manipulated sample (“PLASMID”).

**Fig 3:**
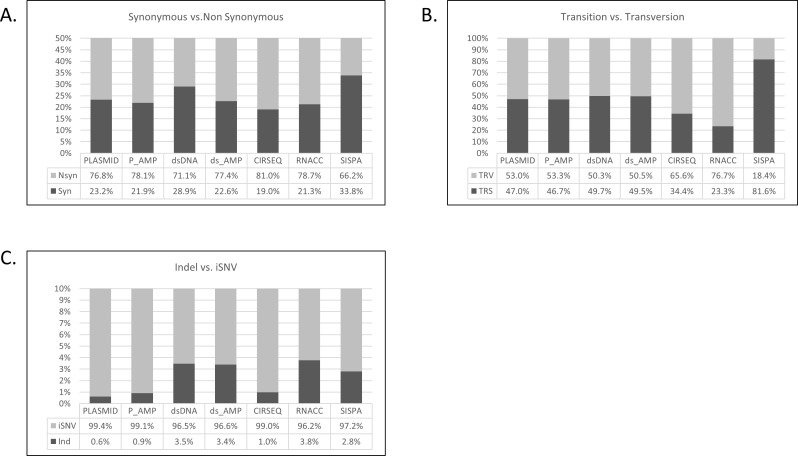
Percentage of errors by type of acquired diversity determined during sample preparation. (A) Percentage of error attributed to synonymous vs. non-synonymous variants. (B) Percentage of errors attributed to transitions vs. transversions. (C) Percentage of errors attributed to insertions or deletions vs. Intra-host single nucleotide variants (iSNV).

We then examined the type and character of the acquired diversity (Fig 3). We stratified the percent of error attributed to synonymous and non-synonymous substitutions (Fig 3A). A mean 24.5% of the iSNVs were attributed to synonymous changes in all libraries with only minor variation except “SISPA”, which demonstrated a significant increase, 9.3% above the mean (Student T-Test, p = 0.00001). We then identified transition and transversion substitutions (Fig 3B). A mean 47.4% of iSNVs were attributed to transitions. Significant reductions from the mean in transitions were observed for both “RNACC” and “CIRSEQ”, 12.8% (T-Test, p = 0.0167) and 24.8% (T-Test, p = 0.00026), respectively. A significant 34.2% increase in transitions was noted for “SISPA” (T-Test, p = 0.00001). Next, we evaluated the percentage of indels in comparison to substitutions (Fig 3C). A mean of 2.28% of iSNVs were attributed to indels. Minor increases (≈1.1%) above the mean in indels were observed with “dsDNA,” “DS_AMP,” “RNACC,” and “SISPA”. In contrast, a ≈1.4% decrease from the mean was observed for “P_AMP”, “CIRSEQ” and “PLASMID”. When these groupings are compared, a 2% significant increase (T-Test, p = 0.00001) in indel iSNVs were observed in protocols with non-correcting reverse transcription steps, suggesting that these indels are acquired or fixed during transcription and reverse transcription.

**Fig 4:**
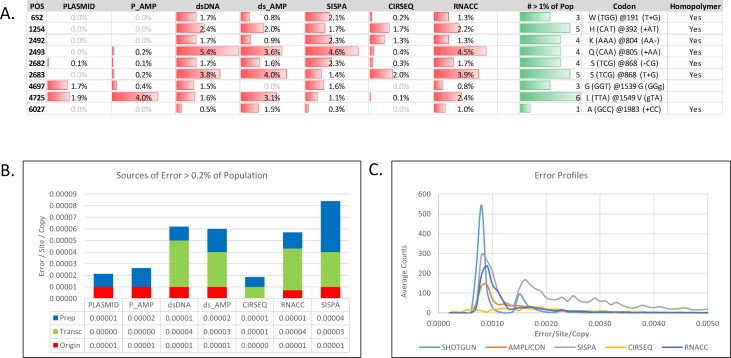
Sources of error per sample preparation method. (A) Intra-host single nucleotide variants (iSNVs) that were present in multiple samples at greater than 1% of population are shown in a heat map format to visualize patterned diversity acquired during sample preparation. The total number of samples containing iSNVs in greater than 1% of population are summarized in the column “#> 1% of Pop” in green. “Codon” column (second column from right) provides both the nucleotide and protein translation of the site. (B) The detected mean error rates of iSNVs greater than 0. 2% of population (error/site/copy) are stratified by presence in the plasmid (Origin), detection after transcription/reverse transcription (Transc) or preparation, and preparation/sequencer error (Prep). (C) Error profiles are expressed as the number of iSNVs per percent of population obtained from each of the 5 sample preparation methods.

To evaluate patterns of individual iSNVs, we used a cutoff of 1% supported in multiple samples. Fig 4A provides an error heat map based on the average percentage of the affected population between replicates for each sample preparation. Two groups were easily identifiable using these parameters. Originating diversity, which consisted of two substitutions at reference positions 4,697 and 4,725 and the remaining patterned sites, were observed in preparations derived from RNA. All of the well-supported sites following transcription proved to be indels either within or immediately following homopolymer regions. Considering that “CIRSEQ” performed extremely well in reducing these types of errors, indels are likely to be systematic errors of reverse transcription.

Using complex sampling (e.g., longitudinal studies) or post-processing methods, detection of rare iSNVs is possible. To describe potentially significant remaining sample preparation error, we set criteria for preparation/sequencer error (“Prep”) as the mean errors of sites greater than 0.2% of population that were not found in in the plasmid (“Origin”), or post-transcription (“Transc”). Fig 4B provides a summary of mean errors /site/copy of iSNVs stratified by originating diversity present in “Origin”, “Transc” or “Prep”. “CIRSEQ,” using these criteria, performed substantially better in regards to sample preparation error than any other library (8.5^−6^ errors/site/copy), and the impact of transcription/reverse transcription errors was significantly (3–4 fold) lower than any other RNA-derived library. However, “CIRSEQ” resulted in a purifying effect on the originating diversity and eliminated both sites detected by all of the other methods, suggesting sensitivity limits in detecting rare variants. Shotgun sequencing residual error was determined to be 1.2^−5^ errors/site/copy from the mean of the “PLASMID” and “dsDNA” sample residual error. Amplicon amplification of both “P_AMP” and “DS_AMP” libraries resulted in comparable residual preparation errors 1.8^−5^ errors/site/copy and similar originating error. Under these conditions, “RNACC” demonstrated the best conservation of the originating diversity and lowest preparation errors/site/copy of 1.4^−5^ (a larger loading amount of 1 ng [rather than 50 pg] of viral RNA resulted in purification of the originating diversity likely due to exhaustion of the available probes), whereas, SISPA demonstrated the highest preparation error/site/copy of 4.4^−5^ (Fig 4B). The error profile expressed as the number of iSNV positions per percent of population (Fig 4C) demonstrates that the majority of the errors for all methods, except “SISPA”, occur in a narrow distribution around 0.09% of the population.

## Discussion

This study presents the deep sequencing results of a reporting system designed to describe acquisition of subclonal diversity resulting from sample preparation techniques commonly used to describe viral populations. Methods utilized in our viral re-sequencing pipeline software VSALIGN were designed to limit the originating diversity present in the plasmid so that acquired diversity (sample preparation error) can be tracked throughout. These methods were used to evaluate five different direct sequencing and amplification preparation techniques. The data obtained here is considered crucial for regulatory purposes, as low error rates of viral population analysis methods are necessary for the study and detection of viral adaptation during antiviral treatment.

To understand the error rate in the viral population, it is critical to know the errors detected by NGS (the numerator) and the number of viral variants queried in the library preparation (the denominator). Unfortunately, the challenge in some of the common proceedings of viral population amplification is to know the true number of templates queried (i.e., the true sequencing depth). Some methods have been proposed to estimate the number of viral templates queried by NGS (e.g., primer barcodes). Unfortunately none of them are generic enough to be utilized by all the protocols evaluated here. To minimize this shortcoming, our experimental design includes homogeneous DNA or RNA populations as templates, thereby diminishing the effect of sampling. In addition, we present the data in comparison to those acquired with “CIRSEQ”, which resulted in the lowest error sequencing rate, and which due to a unique library preparation process determines the true sequencing depth directly. Given that all other procedures evaluated in our work resulted in a higher error rate, we infer that our process to eliminate duplicates could result in the underestimation of the error sequencing rate, but our conclusions regarding the level, scale and source of those error are not affected. The ability of the “CIRSEQ” protocol to limit reverse transcription and sequence errors is quite striking. However, the loss of detection of the plasmid-originating diversity is a concern in regard to sensitivity. Starting material for CirSeq requires 10^7^ genome copies per ml in the absence of complicating genetic host material. While CirSeq would clearly improve the study of viral populations *in vitro*, this method will have to be improved for analysis of *in vivo* samples in which viral loads frequently do not reach 10^7^ genome copies per ml [26]. In a setting in which very precise measurements of larger subclonal populations are required and viral RNA is abundant, “CIRSEQ” would be ideal. On the other end of the input requirement spectrum, “RNACC” excels at recovering viral sequence from low titer and degraded RNA conditions [3].

Finally, “SISPA” is a common protocol for pathogen discovery applications that should be used conservatively for population genetics due to its high sample preparation error rates and the increased number of transitions events. The abundance of transition events may accumulate, causing incorrect assertions of subclonal diversity or masking real diversity present in the sample from post-processing algorithms. With the intent to survey subclonal populations, RNA Access is the best choice of the methods compared in terms of sensitivity and fidelity for *in vivo* amplicon amplification.
